# Influence of practice location on prescribing, diabetes care, and colorectal cancer screening among Czech general practitioners during the COVID-19 pandemic

**DOI:** 10.4178/epih.e2024033

**Published:** 2024-02-23

**Authors:** Jan Bělobrádek, Luděk Šídlo, Tom Philipp

**Affiliations:** 1 Institute of Preventive Medicine, Charles University Faculty of Medicine in Hradec Králové, Hradec Králové, Czech Republic; 2 Department of Demography and Geodemography, Charles University Faculty of Science, Praha, Czech Republic; 3 General Health Insurance Company (GHIC), Praha, Czech Republic; 4 Charles University, Third Faculty of Medicine, Rheumatology and Rehabilitation Clinic, Praha, Czech Republic

**Keywords:** Primary health care, Rural health, COVID-19, Prescriptions, Diabetes mellitus, Cancer screening tests

## Abstract

**OBJECTIVES:**

The provision of primary health care was not interrupted during the coronavirus disease 2019 (COVID-19) pandemic in Czechia, although the capacity and resources of providers changed. We examined how the pandemic affected individual general practices throughout 2017-2021, focusing on differences between urban and rural practices.

**METHODS:**

We analysed data from the largest health insurance company in Czechia, which provides care to 4.5 million people (60% of the population). We evaluated the prescription volume, diabetes care procedures, and faecal immunochemical test (FIT) in preventive care and new pandemic-related procedures (remote consultations, testing, and vaccinations). For the spatial distribution of practices, we adapted the Organisation for Economic Cooperation and Development typology.

**RESULTS:**

We observed minimal declines in 2020 in the rate of prescribing (-1.0%) and diabetes care (-5.1%), with a rapid resumption in 2021, but a substantial decline in FIT (-17.8% in 2020) with slow resumption. Remote consultations were used by 94% of all practices regardless of location, with testing and vaccinations more commonly performed by rural general practitioners (GPs).

**CONCLUSIONS:**

Primary care in Czechia rose to the challenge of the COVID-19 pandemic, as shown by the finding that the volume of healthcare services provided through primary care did not decrease across most of the monitored parameters. This study also confirmed that rural GPs provide more care in-house, both in terms of prescribing and procedures performed in their practices. Future studies will need to focus on preventive care, which the pandemic has dampened in GP practices in Czechia.

## GRAPHICAL ABSTRACT

[Fig f3-epih-46-e2024033]

## Key Message

Primary care in Czechia has effectively adapted to the changes brought about by the COVID-19 pandemic. Minimal impact was observed in prescribtions and diabetic patient care. There was a significant decline in colorectal cancer screening, with a slow restitution after the pandemic subsided. Rural GPs consistently provided in-house treatment and have higher shares of both prescribing and diabetes care, as well as performing more COVID-19 specific procedures.

## INTRODUCTION

When coronavirus disease 2019 (COVID-19) was declared a pandemic in March 2020 [1], primary healthcare systems around the world had been undergoing unprecedented changes for several weeks [2,3] and were bracing for further transformations in the period ahead [4,5]. Prior recommendations were based solely on models of an influenza pandemic [6,7]. As a result, the full extent of the impact on the economy, social life, and healthcare systems during an actual pandemic like COVID-19 could not have been anticipated. Although primary care providers managed the initial surge of the disease on a global scale, several areas have been identified that will require future attention [8].

The greatest concern remains the fate of patients who delayed their doctor visits during the pandemic for various reasons [9]. This group includes patients with chronic conditions who missed their regular follow-ups, potentially leading to inadequate management of their conditions and all associated negative outcomes, such as increased complications, hospitalisations, and mortality [10]. Additionally, the situation has led to a decline in preventive care, particularly evident in the reduced number of cancer screenings and diagnostic tests [11].

The inappropriate geographic distribution of physicians, which disproportionately affects rural areas, has long been recognised as a global issue in primary care [12]. This challenge is not limited by the size or economic status of European countries [13,14]. Czechia has observed similar trends to those in neighbouring countries [15]. From 2010 to 2019, general practitioners (GPs) in Czechia have seen an increase in the average age of doctors, coupled with a territorially uneven and insufficient renewal of the workforce, with the slowest rates of replenishment occurring in rural regions [16]. These dynamics likely impacted the delivery of care during the COVID-19 pandemic, as the aging and understaffed network of rural GPs came under increased pressure from patients, particularly because they were often the sole healthcare coordinators available in their locations.

While numerous papers have addressed the impact of the COVID-19 pandemic on primary care, emphasising the abrupt changes it necessitated—such as reduced patient contact, operational modifications due to anti-pandemic measures, testing and vaccinations, and the adoption of telemedicine [10,17]—our study sought to provide a more comprehensive perspective on the functioning of GP practices during this period. We focused on exploring how GPs adapted to the new responsibilities, including remote consultations, testing, and vaccinations, while also managing their customary pre-pandemic activities.

The differences between rural and urban GP practices during the COVID-19 pandemic were demonstrated in the general conditions of providing care, as shown in an international comparison [18]. The breadth of the data we had access to enabled us to quantify these potential differences across various activities, improving our understanding of how GP practices functioned during the pandemic. Our goal was to determine whether the introduction of new activities—such as remote consultations, testing, and vaccinations—overwhelmed the capacity of GP practices, potentially displacing the activities that GPs typically performed before the pandemic. Since we had a robust dataset at our disposal, which might not provide sufficiently illustrative aggregate outputs, particularly in an international comparison, we opted to select procedures that we believe are sufficiently representative of individual GP activities. These procedures are also pertinent to primary care and can be easily understood in an international context.

To evaluate the overall volume of healthcare services provided, we focused on the prescription patterns and their distribution between GPs and other doctors working in outpatient care. Despite international comparisons showing no reduction in the prescription of chronic disease medications during the COVID-19 pandemic [19], there was a notable decrease in antibiotic prescriptions [20]. We specifically monitored patients with diabetes receiving chronic care, as their management is a high health priority within the European Union (EU); diabetes accounted for 9% of EU healthcare costs in 2019 [21]. In the realm of preventive oncology care, we focused on the faecal immunochemical test (FIT), also known as the faecal occult blood test. The associated screening program aims to prevent colorectal carcinoma, which is another significant healthcare priority in the EU [22]. This concern is relevant to the entire population, unlike breast and cervical cancers.

Concerning activities related to COVID-19, we assessed the frequency of remote consultations, the number of diagnostic tests administered (only antigen tests were conducted in GP practices in Czechia), and the administration of vaccinations. Given the phased implementation of these procedures, a comparison of individual pandemic years was not meaningful. In 2020, only a limited number of tests were carried out, and vaccinations commenced in centrally established vaccination centres. It was not until 2021 that the full capacity of GP practices was utilised for all the aforementioned activities.

To categorise individual practices along the urban-rural spectrum, we employed a typology of general practices that we developed, drawing on the Organisation for Economic Cooperation and Development (OECD) typology, which has been consistently utilised in the authors’ prior research. To reduce errors in our assessment that could arise from overall changes in healthcare services, we selected a 5-year monitoring period (2017-2021). Within this timeframe, the initial 3 years were considered representative of typical conditions, while the latter 2 years were influenced by the COVID-19 pandemic.

## MATERIALS AND METHODS

Our data were obtained from the largest health insurance company in Czechia, the General Health Insurance Company of the Czech Republic (GHIC). During the period under review, the GHIC covered 5.9 million insured individuals, including 4.5 million adults, which represents approximately 60% of the Czech population [23]. Nearly all healthcare providers in Czechia have contracts with this health insurance company.

We used anonymised data related to individual providers, identified by facility ID (as providers in Czechia may operate multiple practices), organised monthly for the years 2017-2021 [24]. Prescribing data was further divided into prescriptions written by GPs themselves and those written by other physicians in outpatient care, such as specialists and specialist hospital outpatient departments. For diabetes care, we evaluated the management of uncomplicated type 2 diabetes mellitus patients who were registered with practices by personal choice. It is important to note that GPs in Czechia do not manage type 1 diabetes mellitus or complicated type 2 diabetes mellitus patients—that is, those typically treated with incretins, sodium-glucose linked transporter 2 (SGLT2) inhibitors, thiazolidinediones, acarbose, glinides, and insulin—as their care is referred to specialists. Patient health checks adhere to the recommended frequency of 4 times per year. In terms of cancer screening, we analysed the outcomes of the “signal procedure–negative test” and the “signal procedure–positive test.” These procedures are automatically included in the contracts of all GP practices in Czechia, as the law mandates the performance of these screenings. Over the review period, there were changes in the testing methodology and the procedures reported. Consequently, the signal codes were the only reliable measure for evaluation.

We assessed procedures directly linked to COVID-19 within the overall totals, taking no account of the fluctuations in legislative changes or the progressive development of the contractual framework with healthcare payers. Remote consultations emerged as a newly defined procedure, whereas testing primarily consisted of administering antigen tests (in Czechia, GPs were reimbursed solely for antigen tests, while specialised centres carried out polymerase chain reaction [PCR] tests). Regarding vaccinations, we tracked the total number of vaccine administrations, encompassing all vaccine types and including both initial vaccinations and booster doses.

To categorise practices along the urban-rural spectrum, we employed a methodology previously described and utilised in other studies [16,25]. Following the OECD guidelines, we classified general practices into 3 categories: urban, intermediate, and rural. Another criterion for evaluation is the availability of a hospital offering acute care in at least 1 core specialty (internal medicine, surgery, paediatrics, or gynaecology) within the settlement. This categorisation is conducted at the level of municipalities with extended powers, which are smaller towns in Czechia that hold a regional level of competence in state administration. Consequently, the intermediate category was further divided into 2 subcategories: municipalities with extended powers that have such a hospital and those without. If a provider operates multiple practices across different categories, they were assigned to the category where they have the most contracted working hours. Table 1 displays the total number of practices for each year monitored, along with the proportion of each category.

## RESULTS

**Figure 1.**
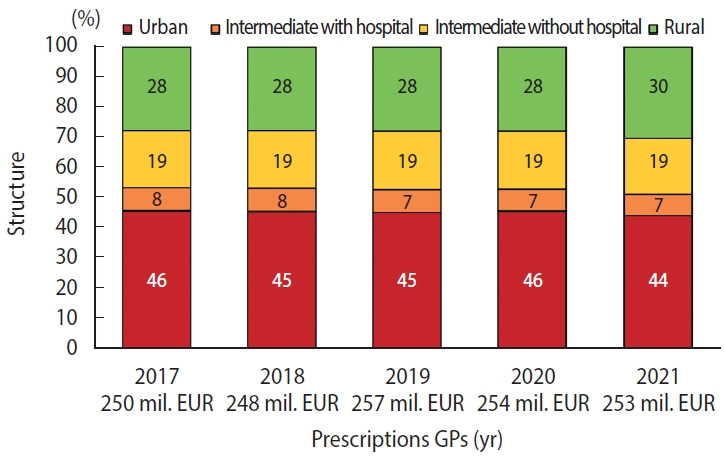
Structure of prescriptions by general practitioners (GPs) through the General Health Insurance Company of the Czech Republic [24]. mil. EUR, million euro.

Supplementary Material 1 presents the compiled data on prescription trends. The consistent annual increase in outpatient prescribing was disrupted in 2020 with a 1.0% decrease. However, in 2021, prescribing resumed its upward trajectory with a 3.2% increase. A comparison of different practice types revealed a slight decline in urban practices and a slight increase in rural practices. The distribution of these types aligned closely with the overall representation of general practices (Table 1). Specifically for GP prescribing, there was a notable shift in proportion favouring rural practices (Figure 1). This aligns with previous research indicating that rural general practices in Czechia tend to prescribe more medications in-house than their urban counterparts [28].

**Figure 2.**
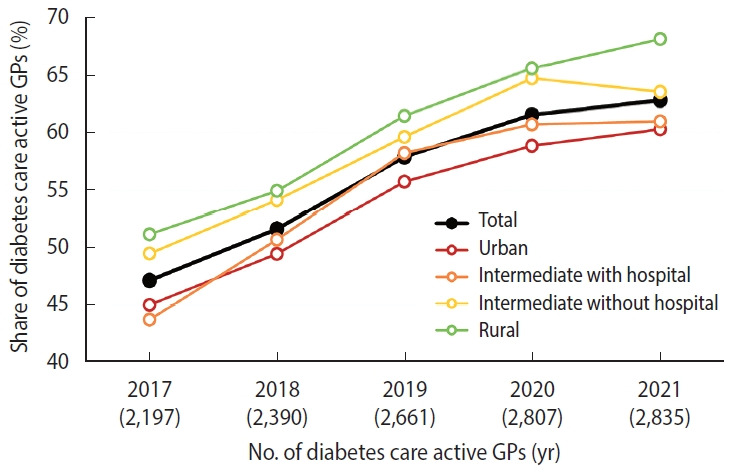
Diabetes care: share of diabetes care by active general practitioners (GPs) by urban-rural typology, General Health Insurance Company of the Czech Republic [24].

Supplementary Material 2 presents data on GP care for patients with diabetes. Figure 2 illustrates a steady rise in the number of practices offering care for diabetes patients, a trend that persisted despite the COVID-19 pandemic. In 2020, there was a 5.1% reduction in the number of treatments; however, the volume of treatments rebounded to the 2019 levels by 2021. Although there was an increase in the proportion of GPs providing care for patients with diabetes towards the periphery, the distribution among different types of general practices remained relatively unchanged throughout the years under review and was seemingly unaffected by the COVID-19 pandemic.

We observed different dynamics for colorectal cancer screening (Table 2). Prior to the COVID-19 pandemic, there was a slight increase in the total number of tests performed. However, there was a notable 17.8% decrease in 2020. This decline was solely due to a reduction in negative tests, which fell by 20.6% that year. Conversely, the number of positive tests actually increased, with a seemingly paradoxical peak in 2020, showing the highest year-on-year increase at 17.1%. In 2021, the volume of tests administered rebounded by 13%, yet the total remained comparable to the figures from 2017. Notably, the incidence of positive tests in 2021 was 60% higher than that observed in 2017.

The specific procedures related to COVID-19 are provided in Table 3. They include remote consultations, tests, and vaccinations aggregated over the years 2020 and 2021. There was a notably high utilisation of remote consultations, with over 94% of GP practices employing this method. The distribution of these consultations did not vary by practice type when compared to the total number of practices. Regarding testing, rural practices conducted a greater number of tests than their urban counterparts (44.2 vs. 30.7%), despite representing only half as many practices. Similarly, rural practices were more prominently represented in vaccination efforts, a trend that was also supported by intermediate-type practices without hospitals, which displayed characteristics akin to those of rural practices. The overall participation of general practices in administering COVID-19 vaccinations was nearly 86%.

## DISCUSSION

We consider the data from the largest health insurer in Czechia to be sufficiently representative due to its robustness and nationwide coverage, despite the variation in the share of the insured across different regions. This variation could primarily affect quantitative indicators. However, because our comparison did not focus on individual regions but rather on types of practices within a relatively stable network over time, and because we conducted longitudinal monitoring of the same indicators across consecutive years, we do not view the uneven territorial density of the insured as a methodological barrier in this type of comparison.

At the same time, we have chosen a subset of indicators from the data, which include the total costs of outpatient care for insured patients. This encompassed services provided by GPs and specialists, such as procedures, prescriptions, laboratory tests, diagnostic radiography, home care, capitation, and bonuses, amounting to approximately EUR 2.5 billion per year. These indicators are representative of the care provided. Assessing the total costs at the individual practice level is challenging due to their inherent disproportionality. The indicators we selected are specific to GPs and are uniformly utilised across all practices in Czechia. This is in contrast to certain instrumental procedures, such as electrocardiography (ECG) and point-of-care testing (POCT), which are adopted voluntarily by practices and are not uniformly distributed [26].

Changes in the number of treatments during the COVID-19 pandemic, including economic indicators, have been studied in several European countries. These studies have consistently reported a decrease in in-person patient visits to medical practices, with a shift towards remote consultations. However, the time frames examined were often brief, such as the years 2019-2020 in Catalonia [27] and France [28]. In France, for example, there was a notably greater reduction in specialist activities compared to those of GPs [29]. A study conducted in Poland also had a narrow focus, highlighting the shortcomings of the capitation reimbursement system in adapting to pandemic conditions [30]. Our findings indicate a minimal decrease in total outpatient prescribing in 2020, suggesting that the care for chronic patients was not significantly compromised. We did not observe any substantial changes in the ratio of GP prescribing (consistently one-quarter) to other outpatient prescribing (consistently three-quarters) over the long term. The proportion of GP prescribing has been on a slight decline over an extended period. The COVID-19 pandemic appears to have had no impact on this long-standing trend.

In Canada, there was a reported decrease in the number of visits by patients with diabetes and assessments for organ complications [31]. Similarly, the United Kingdom experienced a drop in the prescribing rates for individuals with diabetes [32]. The pandemic has also introduced several challenges in the long-term management of these patients. While the reduction in routine health checks at clinics may have protected patients with diabetes from the virus, it also heightened the risk of disrupted comprehensive care. This includes delays in consultations for complications, issues with medication availability and adherence, and decreased physical activity due to lockdowns [33]. A study from the United States examined the total number and types of visits, as well as diabetes care and cancer screenings, in 2020. The study observed a slow and partial recovery to pre-pandemic levels [34]. Our findings indicate a decrease in diabetes treatment in 2020, followed by a swift rebound to the levels seen in 2019. The pandemic did not curb the growing proportion of GP practices providing diabetes care, which is an encouraging development. Moreover, the increased involvement of rural practices in diabetes care aligns with previous research, which found that rural general practices in Czechia tend to offer more comprehensive services in-house compared to their urban counterparts [25].

Concerns regarding the impact of reduced colorectal cancer screenings were highlighted at the onset of the pandemic [35,36]. This downturn was observed on a global scale [37,38]. However, there was also a report of a rapid resumption of screening examinations once restrictions were lifted [39]. Despite this, apprehensions about the disruption of screening programs remained widespread. Experts deliberated on which high-risk patient groups should continue to receive cancer screenings, even amidst a pandemic [40]. Our findings indeed indicate a decline in the total number of tests, but this was solely for negative results; the number of positive tests actually rose on a year-over-year basis during the COVID-19 pandemic. This could be attributed to 2 factors: (1) a shift in the testing method—throughout the period in question, physicians adopted the quantitative POCT with a fixed cutoff, leading to an uptick in positive results starting from 2019; and (2) a more selective approach by GPs during the pandemic. The restoration of testing numbers post-pandemic was considerably slower compared to diabetes care. By 2021, the figures nearly returned to those of 2017.

The COVID-19 pandemic transformed theoretical assumptions about the potential of telemedicine in primary health care into a tangible reality. The initial recommendations for remote patient consultations were promptly introduced [17,41], and they quickly became the standard of care for a wide range of diagnoses, not solely for COVID-19 [42]. Physicians began to recognise the future benefits of telemedicine, while also voicing concerns about patients lacking access to the necessary technology [43]. Notably, primary care physicians had a more positive evaluation of telemedicine’s potential and utilised it more frequently than specialists did. This positive assessment and increased usage were particularly evident among female physicians [44]. In Czechia, remote contact was officially recognised as a medical procedure on September 1, 2020. However, its practical implementation was gradual. Some health insurance companies were slow to adopt it and later imposed restrictions, leading to uncertainties about reporting that discouraged some physicians from using it. Similar to trends in other countries, telephone consultations were much more common than video consultations [45]. Despite these challenges, telemedicine has become established and continues to be used today, albeit with some modifications and limitations on the frequency of use in the list of reimbursed procedures.

The involvement of GPs in testing and vaccination efforts in Czechia was influenced by the overall availability of diagnostic tests and vaccines, as well as by changing legislation, which included repeated mandates for widespread testing. This was further complicated by the gradual implementation of relevant procedures by health insurance companies. Consequently, the role of GPs in these activities was heavily regulated, and the frequent changes in regulations and reimbursement policies led to a reluctance among many physicians to participate. Across the country, many accredited centres conducted PCR tests, while antigen testing was primarily allocated to primary healthcare settings, companies, schools, and the general public. In a similar vein, during the initial vaccine shortage, vaccination centres were the preferred venues for administering shots. It was only later that vaccinations became more commonly available in general practices. The close proximity of dedicated testing and vaccination centres negatively impacted GPs’ willingness to be involved in these activities.

Primary care in Czechia effectively managed the tasks brought on by the COVID-19 pandemic, reflecting many trends observed internationally. It is noteworthy that, with the exception of cancer screening, there was no significant decline in the volume of activities that had been carried out for many years. The sector merely experienced a pause in the continuous growth that had been documented in the years preceding the pandemic. Furthermore, primary care was able to adapt to changes in the organisation of health care services, such as remote consultations, and to incorporate new responsibilities, including testing and vaccinations, brought about by the pandemic.

Of the GP activities we monitored, there was a slight decrease in the treatment of patients with diabetes during the pandemic. However, the number of colorectal cancer screening tests conducted experienced a more pronounced decrease. Although the treatment of diabetes patients rebounded to 2019 levels by 2021, the recovery in colorectal cancer screening procedures was considerably slower. Notably, the number of positive results from colorectal screening tests continued to rise during the pandemic, which was an unexpected outcome. This trend may be partly attributed to the change in screening methodology that was implemented between 2018 and 2019.

The total volume of prescriptions in outpatient care decreased by only 1% in 2020, indicating that the availability of medical care did not significantly deteriorate during the pandemic. The long-term trends in the proportion of prescriptions issued by GPs compared to other physicians, as well as the distribution of prescriptions between urban and rural GPs, were not significantly altered by the COVID-19 pandemic. Rural GPs consistently provided in-house treatment and have higher shares of both prescribing and diabetes care. Furthermore, we demonstrated their increased participation in COVID-19-related procedures, including both testing and vaccinations.

The new procedure of remote consultation was adopted widely among GPs, with a utilisation rate of 94%. This practice has persisted even after adjustments to the conditions, and it continues to be covered by health insurance companies. Looking ahead, it will be essential to monitor how GP activities in Czechia are evolving in response to the COVID-19 pandemic, with particular attention to preventive care and cancer screening programs.

## Figures and Tables

**Figure f3-epih-46-e2024033:**
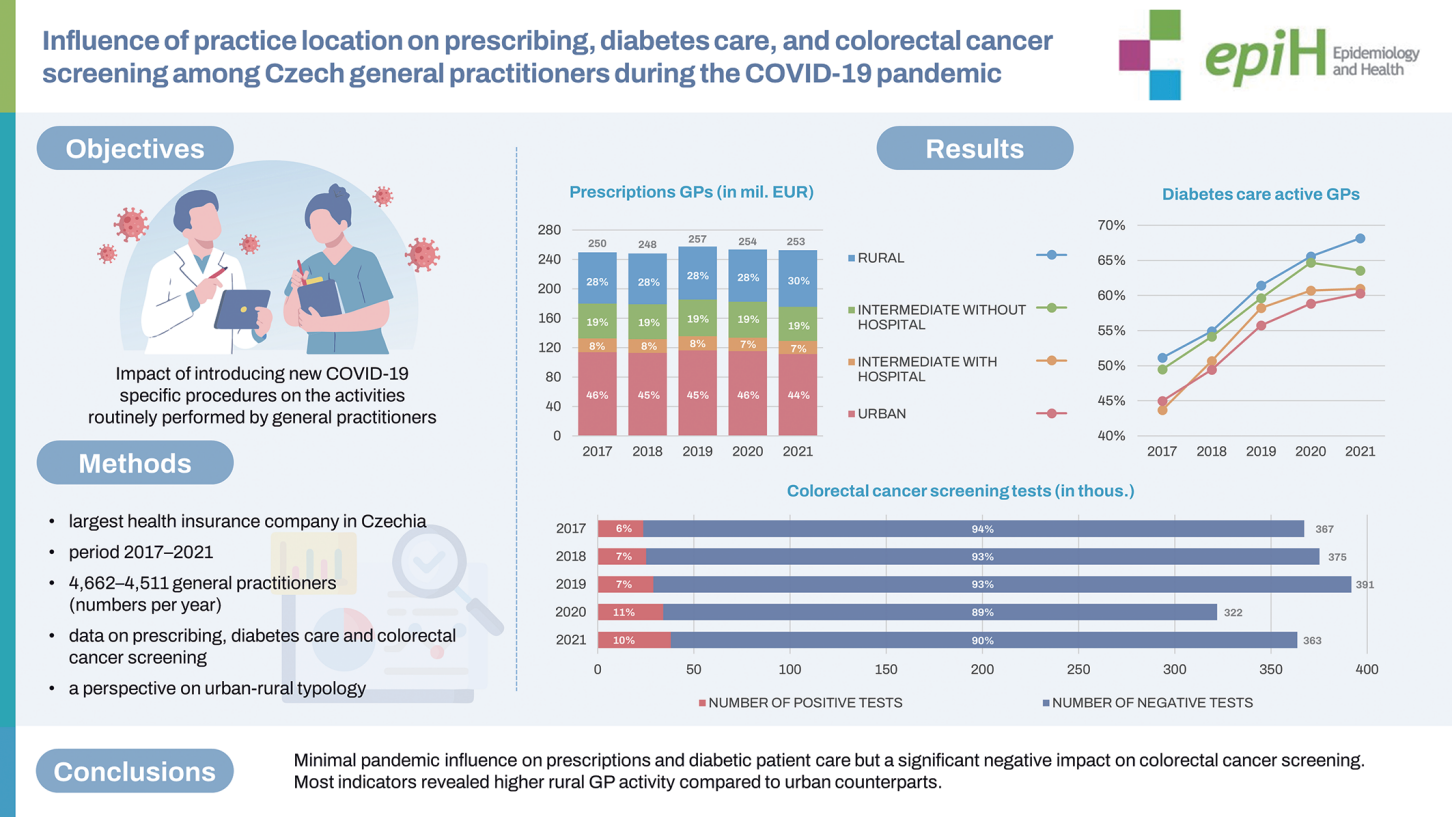


**Table 1.** Number and structure of general practitioner (GP) providers by urban-rural typology^1^

Variables	2017 (n=4,662)	2018 (n=4,630)	2019 (n=4,595)	2020 (n=4,560)	2021 (n=4,511)	Population ≥15 (%)^2^
Urban	2,485 (53.3)	2,469 (53.3)	2,452 (53.4)	2,436 (53.4)	2,370 (52.5)	43.9
Intermediate with hospital	293 (6.3)	288 (6.2)	285 (6.2)	280 (6.1)	269 (6.0)	5.2
Intermediate without hospital	762 (16.3)	761 (16.4)	758 (16.5)	754 (16.6)	738 (16.4)	12.6
Rural	1,122 (24.1)	1,112 (24.1)	1,100 (23.9)	1,090 (23.9)	1,134 (25.1)	38.3

Values are presented as absolute numbers and, in brackets, relative numbers in %.

1 Modified from General Health Insurance Company of the Czech Republic; Unpublished dataset with anonymised data for reported healthcare for GP providers provided for analysis, 2022 [24].

2 Population ≥15=structure of the population aged 15 and over (GPs’ patients), 8.9 million in total (as of 1 July 2019, mid-term).

**Table 2.** Colorectal cancer screening performed by GPs according to GHIC data^1^

Variables	2017	2018	2019	2020	2021
Total no. of tests (n)	367,240	375,270	391,802	321,915	363,740
Year-on-year difference	-	2.2	4.4	-17.8	13.0
No. of positive tests (n)	23,726	25,239	29,027	33,986	37,962
Year-on-year difference	-	6.4	15.0	17.1	11.7
% of total tests	6.5	6.7	7.4	10.6	10.4
Structure by urban-rural typology					
Urban	47.8	48.4	49.3	48.4	46.9
Intermediate with hospital	6.6	6.4	6.7	7.5	7.5
Intermediate without hospital	20.1	20.0	19.2	18.2	17.7
Rural	25.5	25.2	24.8	26.0	27.9
No. of negative tests (n)	343,514	350,031	362,775	287,929	325,778
Year-on-year difference	-	1.9	3.6	-20.6	13.1
% of total tests	93.5	93.3	92.6	89.4	89.6
Structure by urban-rural typology					
Urban	48.7	48.7	48.4	48.7	47.9
Intermediate with hospital	7.1	7.2	7.3	6.8	7.0
Intermediate without hospital	19.0	18.9	18.6	18.7	18.3
Rural	25.2	25.2	25.7	25.8	26.7

Values are presented as %. GP, general practitioner; GHIC, General Health Insurance Company of the Czech Republic.

1 Modified from GHIC; Unpublished dataset with anonymised data for reported healthcare for GP providers provided for analysis, 2022 [24].

**Table 3.** COVID-19-specific procedures performed by GPs according to GHIC data^1^

Consultations/Procedures	Total (n)	Structure by urban-rural typology (%)				Active GPs	% of total GPs
		Urban	Intermediate with hospital	Intermediate without hospital	Rural		
Remote consultations	4,519,070	52.3	6.3	18.1	23.3	4,246	94.1
COVID-19 tests	4,472,316	30.7	10.1	15.0	44.2	3,176	70.4
COVID-19 vaccinations	1,442,640	40.2	5.3	20.0	34.5	3,866	85.7

COVID-19, coronavirus disease 2019; GP, general practitioner; GHIC, General Health Insurance Company of the Czech Republic.

1 Modified from GHIC; Unpublished dataset with anonymised data for reported healthcare for GP providers provided for analysis, 2022 [24].

## Supplementary materials

Supplementary Material 1. Structure of prescriptions, GPs, GHIC

Supplementary Material 2. Diabetes care, GPs, GHIC
