# Supplementary material for: Influence of practice location on prescribing, diabetes care, and colorectal cancer screening among Czech general practitioners during the COVID-19 pandemic
Source: Epidemiol Health. 2024 Feb 23;46:e2024033. doi: 10.4178/epih.e2024033 (PMC11176716; doi:10.4178/epih.e2024033)
Supplement: Supplementary Material 1. — Structure of prescriptions, GPs, GHIC [file epih-46-e2024033-Supplementary-1.docx]

**Supplementary Material 1. Structure of prescriptions, GPs, GHIC**

|  | **2017** | **2018** |  | **2019** | **2020** | **2021** |
| --- | --- | --- | --- | --- | --- | --- |
| Prescriptions total (in mil. EUR) | 979.0 | 1,002.3 |  | 1,045.1 | 1,035.2 | 1,068.3 |
| – year-on-year difference |  | 2.4% |  | 4.3% | -1.0% | 3.2% |
| – structure by urban-rural typology: |  |  |  |  |  |  |
| – urban | 50.9% | 50.8% |  | 50.7% | 50.9% | 49.8% |
| – intermediate with hospital | 7.3% | 7.3% |  | 7.2% | 7.1% | 6.9% |
| – intermediate without hospital | 17.6% | 17.7% |  | 17.9% | 17.8% | 17.4% |
| – rural | 24.2% | 24.2% |  | 24.2% | 24.2% | 25.9% |
| Prescriptions GPs (in mil. EUR) | 249.6 | 248.2 |  | 257.4 | 253.6 | 252.9 |
| – from Prescriptions total | 25.5% | 24.8% |  | 24.6% | 24.5% | 23.7% |
| – structure by urban-rural typology: |  |  |  |  |  |  |
| – urban | 45.6% | 45.4% |  | 45.1% | 45.5% | 44.0% |
| – intermediate with hospital | 7.6% | 7.7% |  | 7.5% | 7.2% | 7.0% |
| – intermediate without hospital | 18.9% | 19.1% |  | 19.4% | 19.3% | 18.5% |
| – rural | 27.9% | 27.8% |  | 28.0% | 28.0% | 30.4% |
| Prescriptions other (in mil. EUR) | 729.4 | 754.1 |  | 787.8 | 781.5 | 815.3 |
| – from Prescriptions total | 74.5% | 75.2% |  | 75.4% | 75.5% | 76.3% |

Note: Prescriptions other = other practices in outpatient care. i.e. specialists and hospital outpatient clinics

Source: [1], authors’ calculations.

**References**

1. General Health Insurance Company of the Czech Republic. Unpublished dataset with anonymised data for reported healthcare for general practitioner providers provided for analysis. Praha:

General Health Insurance Company of the Czech Republic; 2022 (Czech).
