# Supplementary material for: Influence of practice location on prescribing, diabetes care, and colorectal cancer screening among Czech general practitioners during the COVID-19 pandemic
Source: Epidemiol Health. 2024 Feb 23;46:e2024033. doi: 10.4178/epih.e2024033 (PMC11176716; doi:10.4178/epih.e2024033)
Supplement: Supplementary Material 2. — Diabetes care, GPs, GHIC [file epih-46-e2024033-Supplementary-2.docx]

**Supplementary Material 2: Diabetes care, GPs, GHIC**

|  | **2017** | **2018** | **2019** | **2020** | **2021** |
| --- | --- | --- | --- | --- | --- |
| Diabetes care active GPs | 2,197 | 2,390 | 2,661 | 2,807 | 2,835 |
| – year-on-year difference |  | 8.8% | 11.3% | 5.5% | 1.0% |
| – % of total GPs | 47.1% | 51.6% | 57.9% | 61.6% | 62.8% |
| – % by urban-rural typology: |  |  |  |  |  |
| – urban | 45.0% | 49.5% | 55.8% | 58.9% | 60.3% |
| – intermediate with hospital | 43.7% | 50.7% | 58.2% | 60.7% | 61.0% |
| – intermediate without hospital | 49.5% | 54.1% | 59.6% | 64.7% | 63.6% |
| – rural | 51.2% | 54.9% | 61.5% | 65.6% | 68.2% |
| Number of consultations | 212,200 | 234,339 | 270,681 | 256,925 | 274,364 |
| – year-on-year difference |  | 10.4% | 15.5% | -5.1% | 6.8% |
| – structure by urban-rural typology: |  |  |  |  |  |
| – urban | 40.0% | 40.9% | 41.4% | 41.7% | 41.0% |
| – intermediate with hospital | 5.4% | 5.6% | 5.9% | 5.7% | 6.2% |
| – intermediate without hospital | 20.7% | 21.0% | 20.7% | 20.2% | 19.4% |
| – rural | 33.9% | 32.5% | 32.0% | 32.4% | 33.5% |

Source: [1], authors’ calculations

**References**

1. General Health Insurance Company of the Czech Republic. Unpublished dataset with anonymised data for reported healthcare for general practitioner providers provided for analysis. Praha:

General Health Insurance Company of the Czech Republic; 2022 (Czech).
